# Parallel Screening of Wild-Type and Drug-Resistant Targets for Anti-Resistance Neuraminidase Inhibitors

**DOI:** 10.1371/journal.pone.0056704

**Published:** 2013-02-20

**Authors:** Kai-Cheng Hsu, Hui-Chen Hung, Jim-Tong Horng, Ming-Yu Fang, Chun-Yu Chang, Ling-Ting Li, I-Jung Chen, Yun-Chu Chen, Ding-Li Chou, Chun-Wei Chang, Hsing-Pang Hsieh, Jinn-Moon Yang, John T.-A. Hsu

**Affiliations:** 1 Institute of Bioinformatics and Systems Biology, National Chiao Tung University, Hsinchu, Taiwan; 2 Institute of Biotechnology and Pharmaceutical Research, National Health Research Institutes, Miaoli, Taiwan; 3 Department of Biochemistry, Chang Gung University, Taoyuan, Taiwan; 4 Department of Biological Science and Technology, National Chiao Tung University, Hsinchu, Taiwan; 5 Center for Bioinformatics Research, National Chiao Tung University, Hsinchu, Taiwan; National Institute for Viral Disease Control and Prevention, CDC, China

## Abstract

Infection with influenza virus is a major public health problem, causing serious illness and death each year. Emergence of drug-resistant influenza virus strains limits the effectiveness of drug treatment. Importantly, a dual H275Y/I223R mutation detected in the pandemic influenza A 2009 virus strain results in multidrug resistance to current neuraminidase (NA) drugs. Therefore, discovery of new agents for treating multiple drug-resistant (MDR) influenza virus infections is important. Here, we propose a parallel screening strategy that simultaneously screens wild-type (WT) and MDR NAs, and identifies inhibitors matching the subsite characteristics of both NA-binding sites. These may maintain their potency when drug-resistant mutations arise. Initially, we analyzed the subsite of the dual H275Y/I223R NA mutant. Analysis of the site-moiety maps of NA protein structures show that the mutant subsite has a relatively small volume and is highly polar compared with the WT subsite. Moreover, the mutant subsite has a high preference for forming hydrogen-bonding interactions with polar moieties. These changes may drive multidrug resistance. Using this strategy, we identified a new inhibitor, Remazol Brilliant Blue R (RB19, an anthraquinone dye), which inhibited WT NA and MDR NA with IC_50_ values of 3.4 and 4.5 µM, respectively. RB19 comprises a rigid core scaffold and a flexible chain with a large polar moiety. The former interacts with highly conserved residues, decreasing the probability of resistance. The latter forms van der Waals contacts with the WT subsite and yields hydrogen bonds with the mutant subsite by switching the orientation of its flexible side chain. Both scaffolds of RB19 are good starting points for lead optimization. The results reveal a parallel screening strategy for identifying resistance mechanisms and discovering anti-resistance neuraminidase inhibitors. We believe that this strategy may be applied to other diseases with high mutation rates, such as cancer and human immunodeficiency virus type 1.

## Introduction

Influenza virus infection is a major public health problem worldwide [1]–[3]. The swine-origin influenza A virus (S-OIV) was shown to have spread to at least 66 countries since its identification in April 2009 [4]. Influenza is a member of the family Orthomyxoviridae, and it has about 3 serotypes including influenza A, influenza B, and influenza C according to the sequences of nucleoprotein and matrix protein [5]. Among the influenza strains, influenza A causes severe epidemics of respiratory illness each year [4].

Potential anti-influenza drug targets, including viral proteins and host factors, have been previously addressed [5], [6]. Neuraminidase (NA) is a proven drug target for discovery of anti-influenza agents. It is composed of a tetramer of identical subunits that is anchored on the surface of the viral envelope. On host-cell surfaces, NA catalyzes the cleavage of terminal sialic acid residues from carbohydrate moieties to facilitate the release of progeny virions from infected cells [7], [8]. Drugs that inhibit NA, including zanamivir (Relenza) and oseltamivir (Tamiflu), are effective therapeutic agents against influenza viruses [9]–[11]. However, some drug-resistant strains have been reported, including an oseltamivir carboxylate-resistant strain (H275Y in N1 numbering; a tyrosine for histidine substitution at position 275 in NA), a zanamivir-resistant strain (I223R; an arginine for isoleucine substitution at position 223 in NA), and a multiple drug-resistant (MDR) strain with both I223R and H275Y mutations [12]–[16]. Therefore, discovery of the next generation of anti-influenza NA agents is necessary to combat emerging drug-resistant strains.

Due to the extremely low hit rates in our previous screening for NA inhibitors using enzyme-based assays, we propose a parallel screening strategy to overcome problems of NA inhibitor resistance. This strategy simultaneously screens WT and MDR NAs, and selects compounds that match subsite characteristics of both NA binding sites. Conventional screening strategies have focused on WT proteins, and inhibitors have been designed accordingly [17]–[19]. Acquisition of resistant mutant residues in protein-binding sites often precedes the development of drug-resistant strains, most commonly in diseases with high mutation rates, such as influenza virus infection, cancers, and human immunodeficiency virus (HIV) type 1 [20]–[22]. Unlike conventional strategies, parallel screening involves three pivotal steps: 1) characterization of mutation subsites, 2) selection of compounds that are simultaneously complementary to WT and MDR proteins in shape and physico-chemical properties, and 3) bioassay for verification of selected compounds. The goal is to identify inhibitors with maintained activity against drug-resistant strains.

We analyzed the subsite containing the dual H275Y/I223R mutation using site-moiety maps [23]. Our previous works show that site-moiety maps can present moiety preferences and physico-chemical properties of binding sites through several anchors [23], [24]. Each of anchors contains a binding pocket (a part of the binding site) with conserved interacting residues, moiety preferences, and interaction type (electrostatic, hydrogen-bonding, or van der Waals). In addition, site-moiety maps have been successfully applied to the study of ligand-binding mechanisms and to the identification of inhibitors [24]. Using anchor-based analysis, we can observe characteristic changes in the mutation subsite and decipher the mechanisms of drug resistance.

We validated the parallel screening strategy by discovering inhibitors that are active against NAs of both WT and MDR strains. Because the I223R/H275Y dual mutation affects the activities of current drugs including zanamivir, oseltamivir, and peramivir, discovering new inhibitors is critical to treatment of the MDR strain. Using the parallel screening strategy, we first found that the subsite with the dual mutation has many differences in volume, polarity, and moiety preferences as compared with the WT subsite. These differences may confer resistance to current drugs. Subsequently, we identified Remazol Brilliant Blue R that is active against WT and MDR NAs. These results demonstrate the utility of this parallel screening strategy in understanding resistance mechanisms and identifying new inhibitors of MDR NA. We believe that this strategy provides a great development in the treatment of other human diseases and drug-resistant pathogens.

## Results

### Overview of the Parallel Screening Strategy

**Figure 1:**
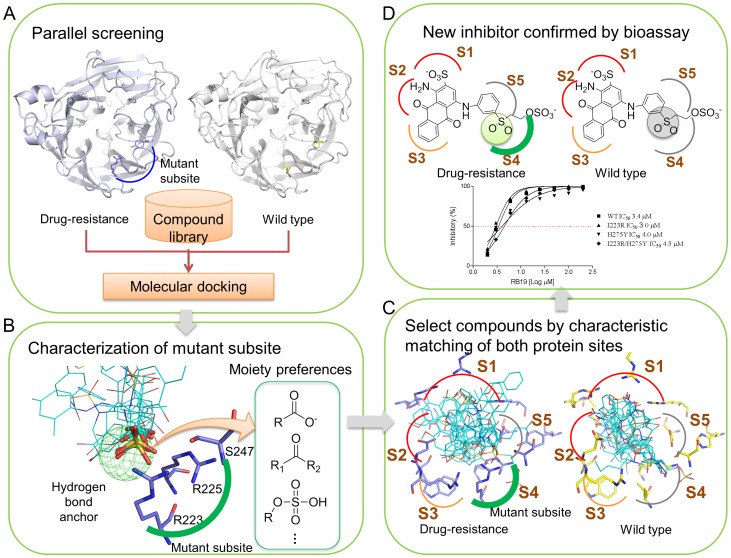
Flowchart of the parallel screening strategy. (A) Parallel screening against WT and MDR NAs. Compounds of the compound library were docked into both NAs using GEMDOCK. (B) Characterization of the mutant subsite by site-moiety map analysis. In the NA site-moiety map, the hydrogen-bonding anchor (colored green) represents a polar environment interacting with polar moieties. (C) Selection of potential anti-resistance inhibitors. Compounds that simultaneously matched characteristics of the 5 subsites for the WT and MDR NAs were selected. (D) Bioassay for verifying the effects of selected compounds on WT and MDR NAs.

Figure 1 shows the framework of the parallel screening strategy for identifying inhibitors of WT and MDR NAs. First, we docked 257,275 compounds selected from public compound databases to binding sites of WT and MDR NAs using our in-house docking tool, GEMDOCK [25] (Fig. 1A). Our previous studies revealed that the performance of GEMDOCK is similar to other docking methods such as DOCK [26], FlexX [27], and GOLD [25], [28], [29]. Furthermore, GEMDOCK has been successfully applied to identify novel inhibitors and binding sites for several targets [30]–[32]. After the docking procedure, we used the docked compounds to characterize the mutant subsite using site-moiety maps [23], which present the relationship between moiety preferences and physico-chemical properties of the binding site through anchors (Fig. 1B).

We divided the binding site into five sub-sites including S1 (R118, R293, and R368 in N1 numbering), S2 (E119, D151, W179, and E228), S3 (R152, W179, and I223), S4 (I223, R225, and S247), and S5 (S247 and E277) based on previous studies [33], [34]. Subsite characteristics of WT NA were described previously [33]. Briefly, by combining the characteristics of the mutated subsite, we selected compounds that simultaneously match characteristics of WT and MDR subsites (Fig. 1C). Finally, the selected compounds were verified by bioassay of WT and MDR NA enzyme activity in the presence and absence of test compounds (Fig. 1D).

### Characterization of Mutant Subsite

**Figure 2:**
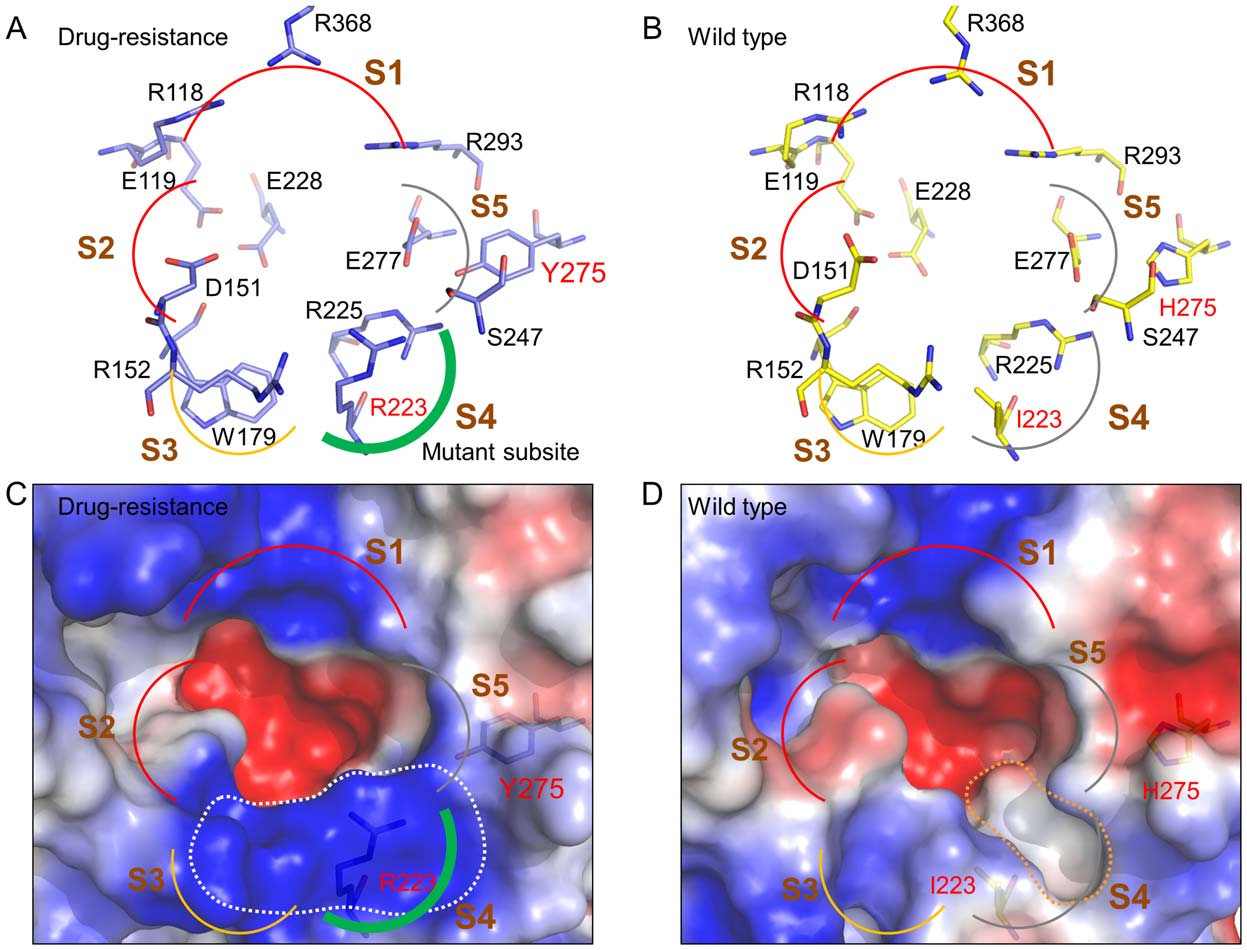
Characteristic comparison of WT and MDR NAs. Binding site residues of (A) MDR and (B) WT NAs. The binding site was divided into the 5 subsites S1 (R118, R293, and R368), S2 (E119, D151, W179, and E228), S3 (R152, W179, and I223), S4 (I223, R225, and S247), and S5 (S247 and E277). The negative/positive, polar, hydrophobic, and mixed hydrophobic and polar subsites are shown as red, green, gray, and orange curves, respectively. These residues are shown in N1 numbering. Molecular surfaces represented by electrostatic potentials of (C) MDR and (D) WT NAs. The negative, positive, and neutral/hydrophobic potentials are colored red, blue, and white, respectively.

Structure comparison of WT and MDR NAs shows striking differences in volume and polarity of S4 subsites, which are mainly caused by the I223R mutation (Fig. 2). Because of the long side-chain of arginine, the volume of the S4 subsite is reduced when I223 is substituted by R223. Furthermore, in the mutant subsite, the residue R223 and its neighboring arginines (R152 and R225) form a positively-charged region (Fig. 2C), whereas the WT S4 subsite is hydrophobic (Fig. 2D) [33], [35]. The reduced volume and changed characteristic of the S4 subsite suggest that inhibitors containing long lipophilic side chains (*e.g.*, oseltamivir) or aromatic rings (*e.g.*, carbocyclic analogue 53 [36]) are inappropriate as inhibitors of MDR NA.

**Figure 3:**
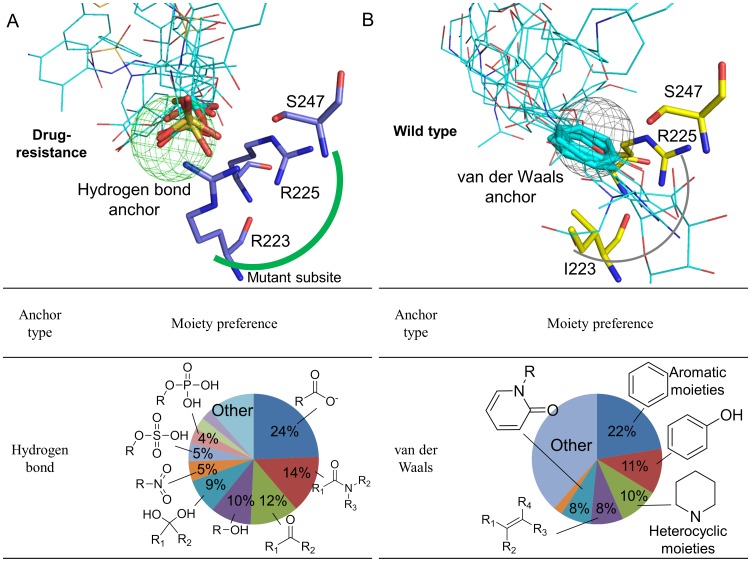
Residues, moiety preferences, and interaction types of anchors in (A) the mutant subsite and (B) the WT subsite. Anchors contain conserved interacting residues, moiety preferences, and interaction types. The hydrogen-bonding anchor (green) indicates that the mutant subsite is polar and prefers to form hydrogen bonds with polar moieties. In contrast, the WT subsite has a van der Waals anchor (gray).

Site-moiety map analyses revealed that a hydrogen-bonding anchor is located at the mutant S4 subsite (R223, R225, and S247) (Fig. 3). The anchor prefers polar moieties such as carboxylic acid, amide, ketone, and sulfuric acid. In contrast, the anchor-type located at the WT subsite is van der Waals interaction, and this anchor prefers ring moieties such as aromatic, phenyl, heterocyclic, and alkene. The difference in moiety preference may be caused by reduced volume and positive charge of the region. Based on these observations, we assumed that inhibitors with large polar moieties (*e.g.*, sulfuric acid derivatives and phosphoric acid derivatives) on the S4 subsite may imply a useful design for maintaining activity against both WT and MDR NAs. Such moieties are able to provide van der Waals contacts with the WT subsite. Moreover, when the dual mutation arises, these moieties may yield hydrogen-bonding interactions with the polar environment.

### Identification of Anti-resistance Inhibitors

We selected compounds that simultaneously fit into characteristics of the binding sites of WT and MDR NAs based on interaction matching and shape complementarity. Subsequently, these compounds were evaluated for their anti-NA activity. The binding sites were divided into 5 subsites (S1–S5) as defined by Stoll *et al*. [33] (Fig. 2). The S1 subsite (R118, R293, and R368) is a positively-charged region, and many inhibitors such as zanamivir and oseltamivir carboxylate (GS4071) interact with this subsite through carboxylic acid moieties [36]. The S2 subsite is composed of residues E119, D151, W179, and E228 and is a negatively-charged environment that interacts with the guanidine of zanamivir through hydrogen bonds. The three residues R152, W179, and I223 of the S3 site possess long side-chains. The crystal structures of protein-compound complexes (PDB codes 3B7E [37], 2HU4 [38], and 1MWE [39]) indicate that the acetamido moieties of sialic acid, zanamivir, and GS4071 consistently form hydrogen bonds with R152 of the subsite. The S4 (I223, R225, and S247) and S5 (S247 and E277) subsites of WT NA are hydrophobic. van der Waals interactions between the two subsites and GS4071 are essential for binding of this inhibitor [40]. It should be noted that the S4 subsite environment changes from hydrophobic to polar when the dual mutation arises. Because these subsites play important roles for NA inhibitor binding, compounds simultaneously interacting with the subsites of the WT and MDR NAs were considered as potential anti-resistance inhibitors.

**Figure 4:**
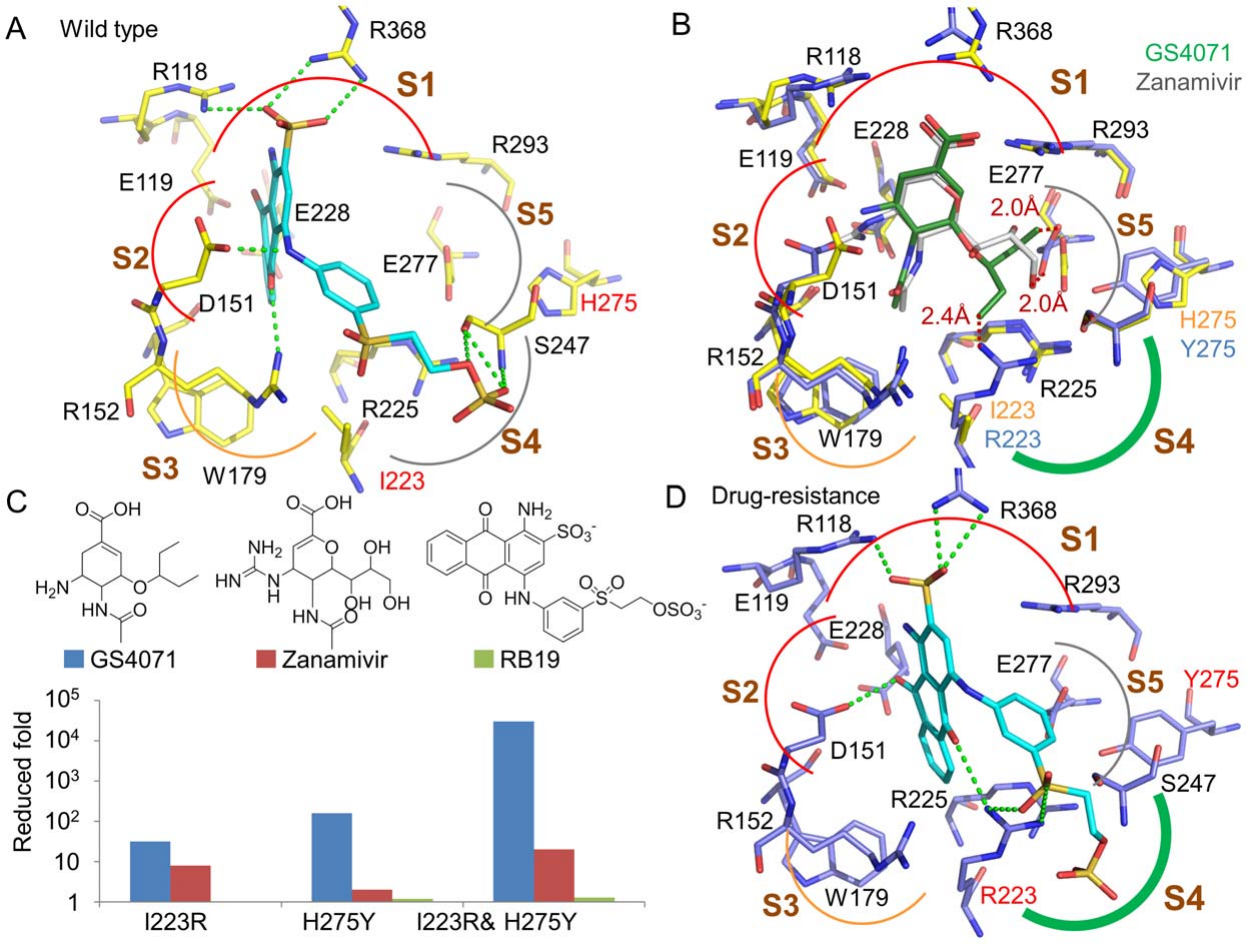
Comparison of binding conformations of RB19, zanamivir, and GS4071. (A) Docking conformation of RB19 on WT NA with hydrogen-bonding interactions represented as light green dashes. (B) Conformations of GS4071 (dark green) and zanamivir (white) in WT (yellow) and MDR (purple) NAs. The GS4071 and zanamivir conformations were derived by superimposing N1 crystal structures (PDB code 3B7E [37] and 2HU4 [67]). (C) Fold changes in IC_50_ of RB19, zanamivir, and GS4071 when the dual H275Y/I223R mutation arises. (D) Docked conformation of RB19 on MDR NA.

Using parallel matching scores (see Methods), we identified Remazol Brilliant Blue R (RB19, an anthraquinone dye) as an anti-resistance inhibitor that was active against both WT and MDR NAs. This compound inhibited the NA of influenza NIBRG14 (H5N1) with an IC_50_ value of 5.7 µM (Table 1), and its docking conformation reveals similar interactions with the 5 subsites as those of zanamivir and GS4071 (Figs. 4A and 4B). The sulfonate moiety of RB19, which has similar physico-chemical properties to the carboxylic acid moieties of zanamivir and GS4071, forms electrostatic interactions with R118 and R368 in the S1 subsite. The electrostatic interactions between negatively-charged moieties and positively-charged residues are consistent with NA complexed with known ligands including sialic acid, zanamivir, and GS4071 (PDB codes 3B7E [37], 2HU4 [38], and 1MWE [39]). In the S2 subsite, the dimethylamine of RB19 yields a hydrogen-bonding interaction with D151, which plays a role similar to that of the guanidine group of zanamivir. However, the inhibitory activity of RB19 is less than that of zanamivir because the guanidine moiety provides six hydrogen-bonding interactions with the residues E119, D151, W179, and E228 in the S2 subsite. These data suggest that addition of a guanidine moiety may increase RB19 potency.

**Table 1:** Inhibitions of RB19, GS4071, and zanamivir on wild-type and mutant NAs.

	IC_50_ (nM) (fold change)^b^		
NA type^a^	RB19	GS4071	Zanamivir
NA^WT^	5.70×10^3^	0.13	0.65
Bac-NA^WT^	3.4×10^3^	0.26	0.54
Bac-NA^I223R^	3.0×10^3^	8.22	4.28
Bac-NA^H275Y^	4.0×10^3^	417.2	1.26
Bac-NA^H275Y&I223R^	4.5×10^3^	7,777.95	10.7

a NA with different mutations. Bac-NA^WT^, Bac-NA^I223R^, Bac-NA^H275Y^, and Bac-NA^H275Y&I223R^ were generated by expressing the wild-type, H275Y, I223R, H275Y and I223R mutants of NIBRG14 (H5N1) NA originating, respectively.

Within the S3 subsite, the ketone on the tetrahydroanthracene moiety of RB19 occupies a similar position to the acetamido moiety of zanamivir and GS4071. This ketone moiety interacts with R152 through a hydrogen bond (Fig. 4A); likewise, the acetamido moieties of zanamivir and GS4071 yield one hydrogen bond with R152. In addition, tetrahydroanthracene makes van der Waals contacts with the long side-chains of residues E117, D151, R152, W179, and E228 of the S2 and S3 subsites. This moiety, which is different to the acetamido group of GS4071 and zanamivir, may be an alternative scaffold for designing NA inhibitors. Similar to the 3-pentyloxy group of GS4071, the sulfone moiety on the aromatic ring of RB19 also forms van der Waals contacts with residues in the S4 subsite (Figs. 4A and 4B). In addition, the sulfuric acid monoester of RB19 forms three hydrogen-bonding interactions with S247 of the S5 subsite.

We further examined the MDR NA inhibitory activity of RB19. In addition, NAs with the respective single mutation (*i.e*., NA^I223R^ and NA^ H275^) were used to evaluate the efficacy of RB19. An insect cell protein expression technology was employed to express these NAs for the study of their sensitivity to RB19 [41]. We first tested GS4071 and zanamivir on these mutant NAs. The experimental results showed that the mutant NA^I223R and H275^, NA^I223R^, and NA^H275^ had 8- to >20,000-fold decreased susceptibility to GS4071, and up to 2- to 36-fold decreased susceptibility to zanamivir (Table 1 and Fig. 4C). In comparison, the IC_50_ values of RB19 for NA^WT^, NA^I223R and H275^, NA^I223R^, and NA^H275^ activity were 3.4, 4.5, 3.0, and 4.0 µM, respectively (Table 1).

The docking conformation of RB19 reveals that two hydrogen-bonding interactions are yielded between the sulfone moiety and R223 of the mutant S4 subsite (Fig. 4D), which may account for the similar inhibition of MDR NA by RB19. The sulfone moiety is able to maintain its interactions with the S4 subsite when the environment changes from a large hydrophobic subsite to a small polar subsite. In contrast, the mutant S4 subsite may not accommodate the 3-pentyloxy group of GS4071 or the glycerol side chain of zanamivir (Fig. 4B). Consequently, these two inhibitors have reduced potency. For GS4071, two clashes are observed between R223 and the 3-pentyloxy group (2.4 Å), and between E277 and the 3-pentyloxy group (2.0 Å). The glycerol moiety of zanamivir is relatively distant from R223, and the hydrogen bonds between the glycerol moiety and E277 may be preserved, leading to a more modest reduction in inhibitory activity. Because of the I223R mutation, the ketone moiety of tetrahydroanthracene switches its hydrogen-bond partner from R152 of the S3 subsite to R223 of the S4 subsite.

The compound RB19 comprises a rigid core scaffold; 1,4-diamino-9,10-dioxoanthracene-2-sulfonate, and a flexible side chain; 2-(3-methylphenyl)sulfonylethyl hydrogen sulfate, both of which are good starting points for designing anti-resistance inhibitors. The core scaffold forms electrostatic, hydrogen-bonding, and van der Waals interactions with the S1, S2, and S3 subsites in both WT and MDR NAs, respectively (Figs. 4A and 4D). Because the residues R118, D151, and R368 of the S1, S2, and S3 subsites are highly conserved in all NA subtypes, and directly interact with the substrate sialic acid [42], [43], mutations on these sites may induce a loss of NA activity. This suggests that the subsites have a decreased probability of acquiring resistance and that the core scaffold is promising for interacting with these conserved regions. Unlike the high conservation of the S1, S2, and S3 subsites, the S4 subsite has relatively low residue conservation and acquires drug resistant mutations such as H275Y and I223R. The 2-(3-methylphenyl)sulfonylethyl hydrogen sulfate moiety has the potential to be applied in the design of anti-resistant drugs because its flexible side chain can tolerate the volume change induced by mutations of S4 residues. The flexible side chain forms van der Waals contacts with the WT S4 subsite. When mutations arise, it changes its orientation to yield hydrogen bonds with the MDR S4 subsite. These interactions maintain the inhibitory activity of RB19, which is similar to that observed in WT NA. These results reveal that RB19 and the two scaffolds are good starting points for the design of new MDR NA inhibitors.

### Interaction Preference of the Mutant Subsite

**Figure 5:**
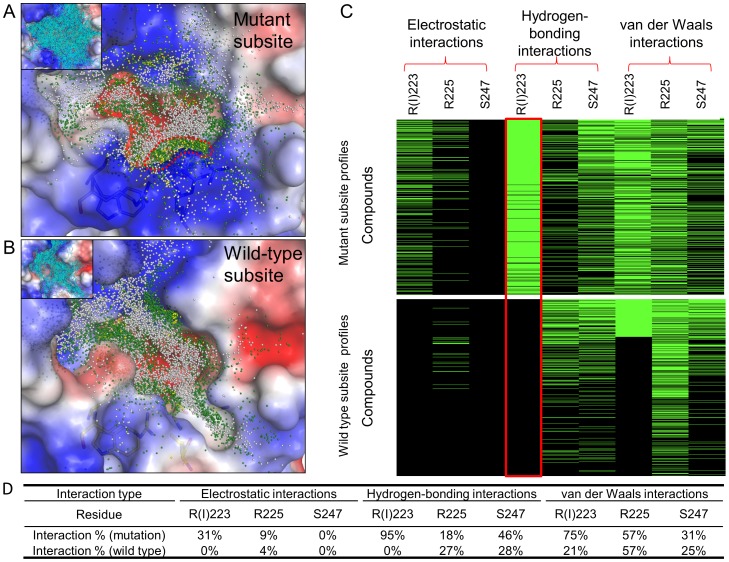
Interaction preference of mutant site. Interacting atom distributions of compounds on (A) MDR and (B) WT NAs. The interacting atoms are shown as grids if interacting with the binding site by electrostatic (yellow), hydrogen-bonding (dark green), and van der Waals (gray) interactions. (C) Protein-compound interaction profiles. An entry is colored green if the screening compound yielded interactions with the residues; conversely, the entry color is black. The red frame shows the major difference of interaction preferences between WT and MDR NAs. (D) Interaction percentages of residues in the profiles.

Understanding interaction preferences of protein subsites can help in the discovery of inhibitors, and in the study of ligand binding mechanisms. To understand the interaction preference of the mutant subsite, we analyzed interaction profiles of the top 600 compounds for WT and MDR NAs using iGEMDOCK [44] (Fig. 5). This tool is a graphical environment used to enhance GEMDOCK for protein-compound interaction visualization and post-screening analysis. For these compounds, their atoms are shown as grids if forming electrostatic (yellow grids), hydrogen-bonding (green grids), or van der Waals (gray grids) interactions with protein residues (Figs. 5A and 5B). The grid distribution reveals that many atoms of the top compounds formed hydrogen bonds with the mutant subsite, whereas the WT subsite has relatively few hydrogen-bonding interactions. The short side-chain of I223 makes a large cavity, resulting in more favorable van der Waals interactions with bulky moieties than the mutant subsite. The strong activity of GS4071 is obtained by optimizing van der Waals interactions with the WT subsite [35].

These interaction profiles show that major differences between the interaction preferences of the two kinds of subsites are dependent on hydrogen-bonding interactions (Figs. 5C and 5D). For example, the MDR subsite residue R223 and the WT subsite residue I223 have 95% and 0% of compounds yielding hydrogen bonds, respectively. The high preference to form hydrogen bonds may account for RB19’s potent activity against the dual-mutant NA. In addition, we found that some compounds have electrostatic interactions with R223 of the mutant subsite, suggesting that designing inhibitors with negatively-charged moieties (*e.g.*, sulfuric acid monoester, phosphonic acid, and carboxylic acid) may enhance potency due to salt-bridge formation.

### Testing the Effect of RB19 using Cell-based Experiments

**Figure 6:**
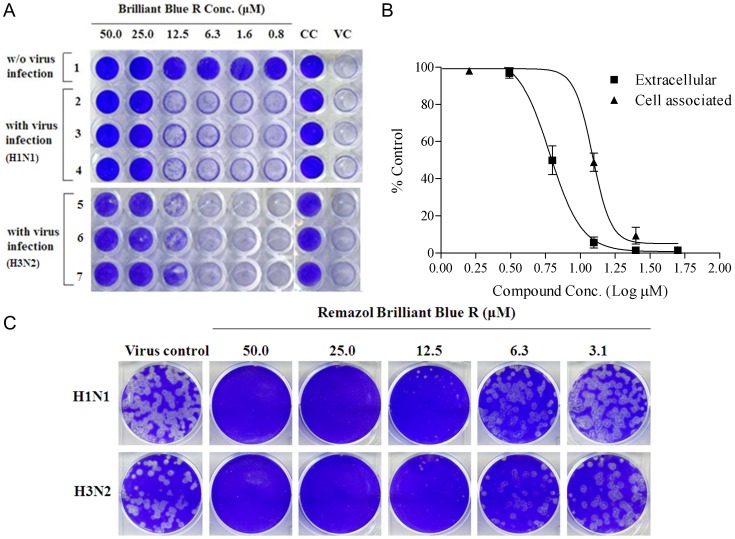
Inhibition of influenza infection and replication by RB19 in MDCK cells. (A) RB19 inhibits the influenza-induced cytopathic effect. In the antiviral neutralization test, MDCK cells were lysed 64 h after A/WSN/33 infection, as shown in the VC (virus control) column. The agent RB19 was added to A/WSN/33-infected cells by two-fold serial dilution starting with a concentration of 50 µM (leftmost column). (B) Reduction in viral yields from infected cells treat w/o RB19 at different concentrations. MDCK cells were infected with MOI 0.001 A/WSN/33 (H1N1) and various concentrations of RB19 were added at the adsorption stage of the A/WSN/33 replication cycle. At 48 h post infection, culture supernatants were collected for virus titration using neuraminidase activity to monitor the viral yield. (C) Inhibition of influenza virus plaque formation by RB19. Approximately 50–100 PFU/well of A/WSN/33 (H1N1) or A/Udorn/72 (H3N2) of influenza A virus was used to infect MDCK cells in 6-well plates. After the viral adsorption stage, 3 ml of agar was overlayed on the media containing various concentrations of RB19. The concentration of RB19 is indicated at the top.

To examine if RB19 inhibits influenza virus replication, we conducted plaque-reduction assays. We used A/WSN/33 (H1N1) and A/Udorn/72 (H3N2) strains instead of the NIBRG14 (H5N1) strain for verification because of limitations of biosafety level in our laboratory. In the plaque reduction assay, the low MOI was used to yield approximately 50–100 plaque forming units per well of cells. Evaluation of RB19 at various concentrations in virus plaque reduction assays was performed for the two different influenza strains. In these experiments, RB19 reduced plaque forming units (PFU) caused by infection of MDCK cells in a dose-dependent manner (Figs. 6A and 6B). The EC_50_ values for viral plaque formation were estimated to be 2.7 µM and 2.8 µM for influenza A/WSN/33 (H1N1) and A/Udorn/72 (H3N2), respectively.

The effects of RB19 on the yields of viral progeny were also examined. Cultured MDCK cells were infected with A/WSN/33 at an MOI of 0.001, followed by the addition of RB19 at various concentrations. The culture supernatants and cell lysates were collected 48 h after infection. Virus titers reflected the NA activity present in the collected samples [45]. Treatment of cells with RB19 dramatically reduced virus yields, as indicated by the fluorogenic signal of NA activity (Fig. 6C). Furthermore, these results demonstrate that higher NA activity was present in cell lysates than in supernatants from RB19-treated infected cell cultures. These results suggest that RB19 treatment may result in the inhibition of influenza virions released into the medium, indicating that NA activity is the major target of this novel anti-influenza compound [45].

Since RB19 is potent in enzyme- and cell-based assays, the anti-influenza activity of RB19 was also examined using two recent clinical isolates, A/TW/70058/09 (H1N1) and A/TW/70066/09 (H1N1), which are resistant to GS4071 [46]. Data from the viral plaque reduction assay shows that EC_50_ values of RB19 on the inhibition of Influenza A/TW/70058/09 (H1N1) and Influenza A/TW/70066/09 (H1N1) replication were 9.8 µM and 11.9 µM, respectively. It is likely that the current drug-resistant problems may be alleviated by novel analogues of RB19.

## Discussion

The potency of inhibitors identified by virtual screening or high-throughput screening is often in the range of micromolar concentration. To further investigate the reasons that RB19 showed micromolar inhibitory activity against NA, we first compared the interactions of RB19 to those of zanamivir, GS4071, and DANA (the first effective NA inhibitor [47], Fig. S1). Based on the subsite interactions between the RB19 and the three compounds, i.e., zanamivir, GS4071, and DANA, we found that the major difference between RB19 and the potent inhibitors (zanamivir and GS4071) are the hydrogen-bonding interactions in the S2 subsite (Fig. S1). RB19 forms one hydrogen bond with the residue D151 (Figs. 4A and S1A), and the IC_50_ value of RB19 is 5.7 µM for the wild-type NA. Similarly, DANA interacts with the S2 subsite with one hydrogen-bonding interaction (Fig. S1B), and inhibits NA with an IC_50_ value 10.0 µM [11]. In contrast, zanamivir (IC_50_ value: 0.13 nM) and GS4071 (IC_50_ value: 0.65 nM) yields six and two hydrogen bonds with the residues of the S2 subsite by the guanidine moiety and the amine moiety (Figs. S1A, S1C and S1D), respectively. According to these results, we will optimize RB19 to achieve low IC_50_ values by increasing the hydrogen-bonding interactions in the S2 subsite in the future.

Based on our parallel screening strategy, we selected seven compounds for bioassay (Fig. S2). Among these compounds, two compounds, RB19 and NSC125899, were considered as the hit compounds. The hit rate of the parallel screening strategy is better than that of the GEMDOCK scoring method (Fig. S3). In this study, the hit rate is low if we directly select compound candidates from the GEMDOCK result because most compounds do not simultaneously match the subsite characteristics of WT and MDR NAs. For example, the ranks of RB19 were 9 and 543 using the parallel matching score and the GEMDOCK score, respectively.

To find the reason that RB19 has better potency than the other compounds, we further analyzed the interactions between the subsites and these compounds (Fig. S4). The interaction profile reveals that the electrostatic interactions at the S1 subsite and the hydrogen-bonding interaction at the residue R368 are essential for compound activities (Fig. S4A). RB19 forms both hydrogen-bonding interactions with the residue R368 and electrostatic interactions with the residues R118 and R368 via its sulfonate moiety (Fig. 4A). In contrast, the compounds with low inhibition percentages (<30%) have no electrostatic interactions in the S1 subsite. For example, NSC674186 and 01502021 lack the negatively-charged groups to interact with positively-charged arginines of the S1 subsite (Figs. S4B and S4C). In addition, van der Waals interactions in the subsites S4 and S5 play the important role for the inhibitor binding. The 2-hydrosulfonylethyl sulfate moiety of RB19 provides additional van der Waals contacts with the residues of the S4 and S5 subsites (Fig. 4A). Conversely, NSC125899 lacks this moiety and shows inhibition percentages of 37% at 20 µM (Fig. S4D).

We selected three RB19 analogues for verifying interactions between their moieties and subsites (Fig. S5). ZINC04016164, which is inactive in inhibition of NA activity, is unable to form van der Waals interactions with the S4 and S5 subsites because it lacks the 2-hydrosulfonylethyl sulfate moiety (Fig. S6A). Similarly, NSC7574 differs from RB19 with two moieties (2-hydrosulfonylethyl sulfate and aromatic ring), and it was inactive for inhibiting NA activity at 40 µM (Fig. S6B). In addition, ZINC04428007 lacks the sulfonate moiety in the S1 subsite and the 2-hydrosulfonylethyl sulfate moiety to form electrostatic interactions with the positively-charged residues of the S1 subsite and van der Waals interactions with the S4 and S5 subsites (Fig. S6C). These results reveal that the importance of the interactions between RB19 and subsites S1, S4, and S5 for inhibiting NA activity.

The parallel screening strategy can be applied to the NAs with different conformation structures. The site-moiety map analysis plays an important role in the parallel screening strategy. It has been successfully applied to elucidate protein-ligand binding mechanisms and enrich the screening accuracy for various proteins such as estrogen receptor, thymidine kinase, Hsp70 protein, epidermal growth factor receptor tyrosine kinase, and shikimate kinase [23], [48], [49]. These results suggest that the parallel screening strategy can be extended to other proteins or NAs with different conformation structure. In addition, we tested RB19 for its utility in inhibiting NA activity of N2, which is phylogenetically distinct to N1. The docking conformations indicated that RB19 has similar interactions with the S4 and S5 subsites of both WT and MDR NAs (Fig. S7). The enzyme-based assay showed that RB19 was able to inhibit N2 NA with an IC_50_ value of 15.3 µM.

The major weakness of virtual screening methods may be the incomplete understandings of ligand binding mechanisms and the subsequently imprecise scoring algorithms [50]–[52]. Our parallel screening and scoring strategy, which combined moiety matching score and energy-based score, was helpful to reduce the deleterious effects of selecting high molecular weight and/or high polar compounds using energy-based scoring functions. Although the parallel screening strategy may not good enough to discover inhibitors in nonamolar concentration against NA, the site-moiety map was able to guide RB19 optimization [23], [24]. In addition, we searched RB19 analogues for improving the potency from two public databases, including PubChem [53] (8,293,922 compounds) and ZINC [54] (17,833,934 compounds). We were unable to find the RB19 analogues that contain a guanidine moiety on the tetrahydroanthracene moiety (Fig. S5). The result indicated that the limited compound databases may be the reason for RB19 presented in much more higher concentrations than GS4071 and zanamivir to inhibit WT NAs. The chemical spaces and bioactivity databases have been investigated for virtual screening methods [53]–[56].

Drug-resistant mutations often emerge during drug therapy and result in treatment failure. For example, the dual mutation of NA, which leads to reduced susceptibility to NA-inhibiting drugs, were detected in patients after treatment with oseltamivir and zanamivir [15], [57]. In another example, gefitinib and erlotinib are often used to target epidermal growth factor receptor for the treatment of non–small-cell lung cancer [58], [59]. Although the drugs are effective, many patients eventually relapse due to resistance mutations [60]. These examples show that designing novel inhibitors with different scaffolds are essential for the treatment of MDR diseases. The parallel screening strategy developed here, which identifies inhibitors for both wild-type and drug-resistant targets, therefore provides a valuable alternative for overcoming such diseases.

Many mutations of NAs have been reported including E119V, D151E, H275Y, R293K, and N295S [42], [57], [61]. Some of these alter the characteristics of the binding site and disrupt drug binding. For example, H275Y, which is a frequent resistance mutation, changes the orientation of E277 and alters the hydrophobic pocket of the S4 subsite, which forms interactions with the 3-pentyloxy moiety of GS4071 [40]. As a result, GS4071 sterically clashes with the altered pocket; dramatically reducing GS4071 activity (Fig. 4B). However, previous studies showed that at least one of the NA drugs usually maintains potency for NAs with the above mutations [62]. The dual I223R/H275Y mutant NA discussed here was relatively impervious to the three traditional NA drugs. Therefore, RB19 and the S4 subsite characteristic are promising starting points for designing inhibitors of MDR strains.

Characteristics of the mutant subsite provide insights for understanding the molecular mechanisms of NA drug resistance. The drug-resistance may be caused by sterically clashes between the mutant S4 subsite and the functional group of GS4071 and zanamivir. GS4071 was designed by optimizing hydrophobic interactions with the wild-type S4 subsite [35]. For the dual mutation I223R and H275Y, the volume of the S4 subsite is reduced because of the long side-chain of arginine and tyrosine (Fig. 2). For GS4071, two clashes are observed between R223 and the 3-pentyloxy group (2.4 Å), and between E277 and the 3-pentyloxy group (2.0 Å) (Fig. 4B). In contrast, RB19 is not affected by some drug-resistant mutations such as I223R, H275Y, and I223R/H275Y NA mutants because RB19 contains a flexible chain with a large polar moiety. This flexible chain forms van der Waals and hydrogen bonds interactions with the wild-type subsite and the mutant subsite, respectively. Therefore, the IC_50_ values of RB19 do not have a big difference between WT and MDR NAs. In addition, large polar moieties (*e.g.*, sulfuric acid derivatives and phosphoric acid derivatives) may be substitutes for the functional groups at the S4 subsite of current NA drugs and may enhance activity against dual-mutant NAs.

To our knowledge, this is the first report that indicates the ability of RB19 to inhibit influenza NA activity. Several dyes have traditionally been known as effective drugs to combat a variety of infectious diseases. For instance, proflavine and acriflavine, an acridine derivative, were used as disinfectant bacteriostatic agents against many *Gram* (+) bacteria [63]. Suramin, a type of azo dye, has been proposed as a therapy for Creutzfeld–Jacob disease [64]. Furthermore, methylene blue has been described for its antimalarial activity [65], [66].

### Conclusion

There is an urgent need for new anti-influenza therapies that target drug-resistant strains. In this study, we developed a novel screening strategy to simultaneously screen compounds that inhibit WT and MDR NAs. Through this strategy, a novel scaffold (RB19) was identified as a good starting lead for designing more effective NA inhibitors that combat oseltamivir-resistant (H275Y), zanamivir-resistant (I223R), and multidrug-resistant (I223R and H275Y) influenza viruses. Further investigations are needed to determine whether current drug resistance can be overcome by developing compounds with the RB19 scaffold. Experimental results demonstrate the utility of this parallel screening strategy in understanding resistance mechanisms and identifying new inhibitors of MDR NA. We believe that this strategy provides a great development in the treatment of other human diseases (such as cancers and HIV type 1) and drug-resistant pathogens.

## Materials and Methods

### Preparation of NA Structures and Screening Library

For the parallel screening strategy, the open-form H5N1 NA structure (PDB code 2HTY [67]) was downloaded from the Protein Data Bank. We selected this structure that is not in complex with ligands because ligand-bound structures may have induced fit and restrict the diversity of identified inhibitors. To define the binding site of NA, the zanamivir-bound structure (PDB code 2HU4 [67]) was aligned to this unbound structure (i.e., 2HTY) using a structural alignment tool [68]. The binding site was defined as residues within a 10 Å radius sphere centered around the zanamivir.

Compound libraries used for virtual screening included databases from the National Cancer Institute (NCI) and Sigma-Aldrich (St. Louis, MO). Compounds were selected for docking if their molecular weights ranged between 200 and 650 daltons and the number of selected compounds was 257,275.

The NA structure with I223R and H275Y dual-point mutations was derived using a homology-modeling server [69]. The protein sequence submitted for the server was from the strain NIBRG14 (H5N1) with the dual-point mutation, which was used to generate MDR NA for the bioassay. The unbound NA structure (i.e., 2HTY) was selected as the structure template. The binding site of the mutant structure was generated using the procedure described above.

### Virtual Screening

Each compound was docked into the binding site of NA using our in-house docking tool, GEMDOCK. This tool rapidly measures intermolecular potential energies between binding sites and compounds using a scoring function that is based on piecewise linear potential [25]. The scoring function of GEMDOCK contains electrostatic, steric, and hydrogen-bonding potentials, and the intermolecular potential energy for protein-compound complexes is calculated as the sum of the three potentials. After docking, candidate compounds were ranked based on their intermolecular potential energy. The top 12,800 (approximately 5%) compounds were docked into the binding site of MDR NA to obtain energy potential rankings.

### Site-moiety Map and Interaction Profile Analyses

The site-moiety map, using docked poses generated by virtual screening tools (e.g. GEMDOCK, GOLD [28], and AutoDock [70]), elucidates protein-ligand binding mechanisms and enriches the screening accuracy for post-screening analysis [23], [48], [49]. From the top compounds, we selected 600 that interact with the S4 subsite and analyzed moiety and interaction preferences of the subsite. These compounds and WT- and MDR NA-binding sites were used to establish site-moiety maps. In theory, at least 500 compounds are required to establish a site-moiety map [23]. First, we used protein-compound interaction profiles to present interactions between compounds and protein residues. Three profile types were used including electrostatic (E), hydrogen-bonding (H), and van der Waals interaction (V) profiles. For each profile, the interactions were represented by a matrix with size *P* × *C*, where *P* and *C* are the compound number and the interacting residue number of the protein, respectively. Interactions were detected using the piecewise linear potential function of GEMDOCK [25]. Profiles were then visualized by iGEMDOCK [44]. The entry of the E and H profiles was set to 1 if there was an electrostatic or hydrogen-bonding interaction between the compound and the residue (green regions in Fig. 5C); otherwise the entry was set to 0 (black regions). For the V profile, entry was set to 1 if its V interaction energy was less than −4 kcal/mol.

We recognized consensus interactions of the profiles using Z-scores as anchors, which often play key roles in biological functions [23], [24]. For each profile, we obtained the Z-score value (*Z_i_*) of the protein residue *i* according to the equation:, 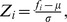 where *f_i_* is the observed interaction frequency between the compounds and the residue *i*, and *μ* and *σ* are the mean and standard deviation of interaction frequency acquired from 1,000 randomly shuffled profiles. Interactions between compounds and the residue *i* with a Z-score ≥1.645, which is a commonly used statistical threshold (95% confidence level), were regarded as consensus interactions. In this way, spatially neighboring residues with consensus interactions and their interacting moieties consisted of an anchor. Here, we used the anchor located at the S4 subsite to characterize the subsites of WT and MDR NAs (Fig. 3).

### Compound Selection for Bioassay

We selected compounds that simultaneously matched the subsite characteristics of WT and MDR NAs because these compounds maintained their potency despite mutations of the enzyme. A compound was considered as matching a subsite if its interacting moiety is physico-chemically complementary to the subsite and if it formed appropriate interactions. For example, the S4 subsites of MDR and WT NAs prefer hydrogen-bonding and van der Waals interactions, respectively, based on the site-moiety map analyses. The sulfone moiety of RB19 forms hydrogen-bonding interactions with the mutant S4 subsite, and RB19 was thereby considered as matching the characteristic of the S4 subsite. The characteristics of S1, S2, S3, and S5 subsites were directly obtained from Stoll *et al*. [33]. The S1 subsite is a positively-charged environment, and the compounds matched the characteristic if yielding electrostatic interactions with the subsite. The S2 subsite environment is negatively charged. Compounds were considered as matching this subsite if they had electrostatic interactions with the environment. For the two subsites, hydrogen-bonding interactions between the compounds and the subsites are also regarded as characteristic matching. The S3 subsite is a mixed hydrophobic and polar region, and hydrogen-bonding or van der Waals interactions were accepted. The S5 subsite is hydrophobic, and the compounds that formed van der Waals contacts were in accordance with this subsite characteristic.

We have developed the parallel matching score, which is an extension of the site-moiety map score [23], to quantify the compound matching both physico-chemical properties of the WT and MDR binding sites. Here, the parallel matching score of the compound *c* is defined as
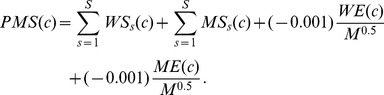
where *WS_s_*(*c*) and *MS_s_*(*c*) are the matching scores of the compound *c* in the subsite *s* of WT and MDR NAs, respectively; *S* is the subsite number; and *WE*(*c*) and *ME*(*c*) are the docked energies of the compound *c* in the WT and MDR NAs, respectively. *WS_s_*(*c*) or *MS_s_*(*c*) is set to 1 if the compound *c* with the moiety matching the physico-chemical properties of the subsite *s*; otherwise, the score is set to 0. The matching scores and the term *M* ^0.5^ are helpful to reduce the deleterious effects of selecting high molecular weight compounds [23], [71] where *M* is the atom number of the compound *c*. These docked compounds were then ranked based on their parallel matching scores. The compound possessing a high parallel matching score is often potential to inhibit both WT and MDR NAs. In order to find compounds that were not affected by dual mutation, compounds were filtered if they did not match the characteristic of the mutant S4 subsite. Finally, the selected compounds that were commercially available were tested using the bioassay.

### Drugs and Reagents

The selected compounds, which were requested or purchased, were dissolved in dimethyl sulfoxide (DMSO) and stored at −20°C. Oseltamivir carboxylate (GS4071) was synthesized by Dr. Kak-Shan Shia at the National Health Research Institutes of Taiwan. The fluorogenic substrate 2′-(4-methylumbelliferyl) -α-d-N- acetylneuraminic acid (MU-NANA) was obtained from Sigma-Aldrich.

### Viruses and Cells

The influenza strains A/WSN/33 (H1N1) and A/Udorn/72 (H3N2) were used in this study [72]. Two clinical isolates that are resistant to GS4071, A/TW/70058/09 (H1N1), and A/TW/70066/09 (H1N1) were utilized in the antiviral assay [46]. Madin–Darby canine kidney (MDCK) cells were obtained from the American Type Culture Collection (Manassas, VA) and were maintained in Dulbecco’s Modified Eagle’s Medium (DMEM) with 10% fetal bovine serum.

### Generation of Oseltamivir- and Zanamivir-resistant NA with H275Y and I223R Point Mutations

The N1 neuraminidase gene for strain NIBRG14 (H5N1) was obtained from our previous study. The recombination plasmid pGEM-T easy, containing the NA gene from strain NIBRG14 (H5N1), was used as a target for I223R site-directed PCR mutagenesis of NA. To construct the I223R and H275Y double mutation expression plasmid, recombinant plasmid pGEM-T easy, containing the single point mutation H275Y NA gene (H5N1), was used as the target for site-directed PCR mutagenesis of NA. To generate I223R-specific mutations, two primer sets were designed as follows: I223R-F; (5′- GAG TTG GAG GAA CAA CAG ACT GAG AAC TCA AGA GTC TG -3′), and I223R-R; (5′- CAG CTC TTG AGT TCT CAG TCT GTT GTT CCT CCA ACT C -3′).

Mutation sites were created by PCR using 0.4 mM dNTP, 0.3 µM primers, 2 ng template DNA, and an appropriate amount of AccuPrimeTM Turbo Pfu DNA polymerase and buffer (Invitrogen, Carlabad, CA). The thermal cycling profile was as follows: 95°C for 5 min, followed by 20 cycles of 94°C for 30 sec, 55°C for 1 min, and 68°C for 8 min, and final extension at 68°C for 7 min. After PCR amplification, DpnI was added to digest the plasmid template. Amplified DpnI-insensitive mutated plasmids were introduced into *Escherichia coli* for selection of the designed mutant sequences.

### Production of NA in Insect Cells

The NA expression constructs were co-transfected, with linear BacPAK8 viral DNA, into Sf9 insect cells as described in a previous report [41]. Culture media from infected cells was then collected and stored as virus stock for the production of NA for the enzyme assay. Recombinant baculoviruses –Bac-NA^WT^, Bac-NA^H275Y^, Bac-NA^I223R^ and Bac-NA^I223R/H275Y^–were generated to express wild-type, H275Y, I223R and I223R/H275Y mutants of NA originating from influenza N1 neuraminidase (NIBRG14 (H5N1)). Total cell lysates were treated with 2.5 mg/ml pronase for 1 hour at 22°C.

### Cell-associated and Cellular Virus Yields

After infection of MDCK cells with MOI 0.001 A/WSN/33, various concentrations of compound were added to the cell media. After 48 h, culture supernatants were taken, and infected cells were scraped off wells and collected by centrifugation at 16000 g for 5 min. To determine the extracellular and cell-associated virus titer after treatment with inhibitors, supernatants and lysates were assayed for neuraminidase activity as described previously [41].

### Evaluation of Antiviral Activities of Neuraminidase Inhibitors

To measure the potential of the identified inhibitors to inhibit cytopathic effects (CPE) in MDCK cells infected with influenza viruses, we used the CPE inhibition assay protocol described by Hung et al. [41]. The concentration of test compound required to reduce the CPE of the virus by 50% (IC_50_) was determined. In these experiments, GS4071 was used as a positive control. Plaque assays were used to determine the effects of inhibitors on influenza virus replication, as described by Hung et al. [41]. The concentration of inhibitors required to reduce the number of plaques by 50% (EC_50_) was then determined. Cell toxicity of inhibitors was determined using an MTS assay [73]. Optical density was measured by ELISA reader at OD490 nm.

### Neuraminidase Inhibition Assay

To inactivate viral infectivity, cell culture suspensions of virus-infected MDCK cells were inactivated with formaldehyde (0.02%) as described by Hung et al [41] and Jonges et al [74]. Enzymatic activity of NA was measured using the fluorogenic substrate MU-NANA, as described previously [75]. To evaluate the inhibitory effects of identified compounds, inactivated virus supernatants were pre-incubated with test compounds for 30 min at 30°C. The assay was conducted in 96-well plates containing diluted virus supernatant (containing active influenza NA) and 100 µM fluorogenic substrate per well in an MES buffer (32.5 mM MES, 4 mM CaCl2 at pH 6.5). The enzyme reactions were then carried out for 1 h at 37°C and were terminated by adding a stop solution containing 25% ethanol and 0.1 M glycine (pH 10.7). Fluorescence intensity of the product 4-MU was measured using a Fluoroskan spectrofluorometer (Labsystems, Helsinki, Finland) with excitation and emission wavelengths of 330 and 445 nm, respectively. The IC_50_ value for NA activity was then determined using GS4071 as a positive control.

## Supporting Information

Figure S1 **Interaction comparison between RB19, DANA, zanamivir, and GS4071.** (A) Interaction profiles between the subsite residues and the compounds. A subsite includes three type interactions (electrostatic (E), hydrogen-bonding (H), and van der Waals (V)). A cell is colored in green if there is interaction (electrostatic, hydrogen-bonding, or van der Waals) between a compound and a residue; otherwise, the cell is colored in black. Binding conformations of (B) DANA (PDB code 1IVF [76]), (C) zanamivir (PDB code 3B7E [37]), and (D) GS4071 (PDB code 2HU4 [67]). The hydrogen-bonding interactions between the compounds and the S2 subsite residues are represented as light green dashes.
(TIF)

Figure S2 **Structures, IC_50_ values, and ranks of the selected compounds.**
(TIF)

Figure S3 **Performance of parallel screening strategy and GEMDOCK scoring method.** RB19 and NSC125899 were considered as the hit compounds for comparing the two scoring methods. The parallel screening strategy has better performance than the GEMDOCK scoring method in identifying the hit compounds. For example, RB19 is ranked as 9 and 543 using the parallel screening method and the GEMDOCK scoring method, respectively.
(TIF)

Figure S4 **Interaction profiles of seven selected compounds.** (A) Interaction profiles between the subsite residues and the compounds. A subsite includes three type interactions (electrostatic (E), hydrogen-bonding (H), and van der Waals (V)) between interaction residues and compounds. A cell is colored in green if a compound forms interaction (electrostatic, hydrogen-bonding, or van der Waals) with a residue; otherwise, the cell is colored in black. Docked conformations of (B) NSC674186, (C) 01502021, and (D) NSC125899 on five subsites.
(TIF)

Figure S5 **Structures and inhibition percentages of RB19 analogues.**
(TIF)

Figure S6 Docking conformations of (A) ZINC04016164, (B) NSC7574, and (C) ZINC04428007 on the wild-type NA of N1.
(TIF)

Figure S7 Docking conformations of RB19 on the (A) wild-type NA (PDB code 2AEQ [77]) and (B) the dual H275Y/I223R NA of N2. The structure with the dual mutation of N2 was generated using the similar procedure as the dual-mutant structure of N1.
(TIF)

Table S1 Structures, IC_50_ values, and ranks of the selected compounds.
(DOC)

Table S2 Structures and IC_50_ values of RB19 analogues.
(DOC)
