# Correction: Parallel Screening of Wild-Type and Drug-Resistant Targets for Anti-Resistance Neuraminidase Inhibitors

**DOI:** 10.1371/journal.pone.0314066

**Published:** 2024-11-13

**Authors:** Kai-Cheng Hsu, Hui-Chen Hung, Jim-Tong Horng, Ming-Yu Fang, Chun-Yu Chang, Ling-Ting Li, I-Jung Chen, Yun-Chu Chen, Ding-Li Chou, Chun-Wei Chang, Hsing-Pang Hsieh, Jinn-Moon Yang, John T.-A. Hsu

The affiliation for the ninth author is incorrect. Ding-Li Chou is not affiliated with #3 but with #4: Department of Biological Science and Technology, National Chiao Tung University, Hsinchu, Taiwan.
