# Supplementary figures and images for: Parallel Screening of Wild-Type and Drug-Resistant Targets for Anti-Resistance Neuraminidase Inhibitors
Source: PLoS One. 2013 Feb 20;8(2):e56704. doi: 10.1371/journal.pone.0056704 (PMC3577712; doi:10.1371/journal.pone.0056704)

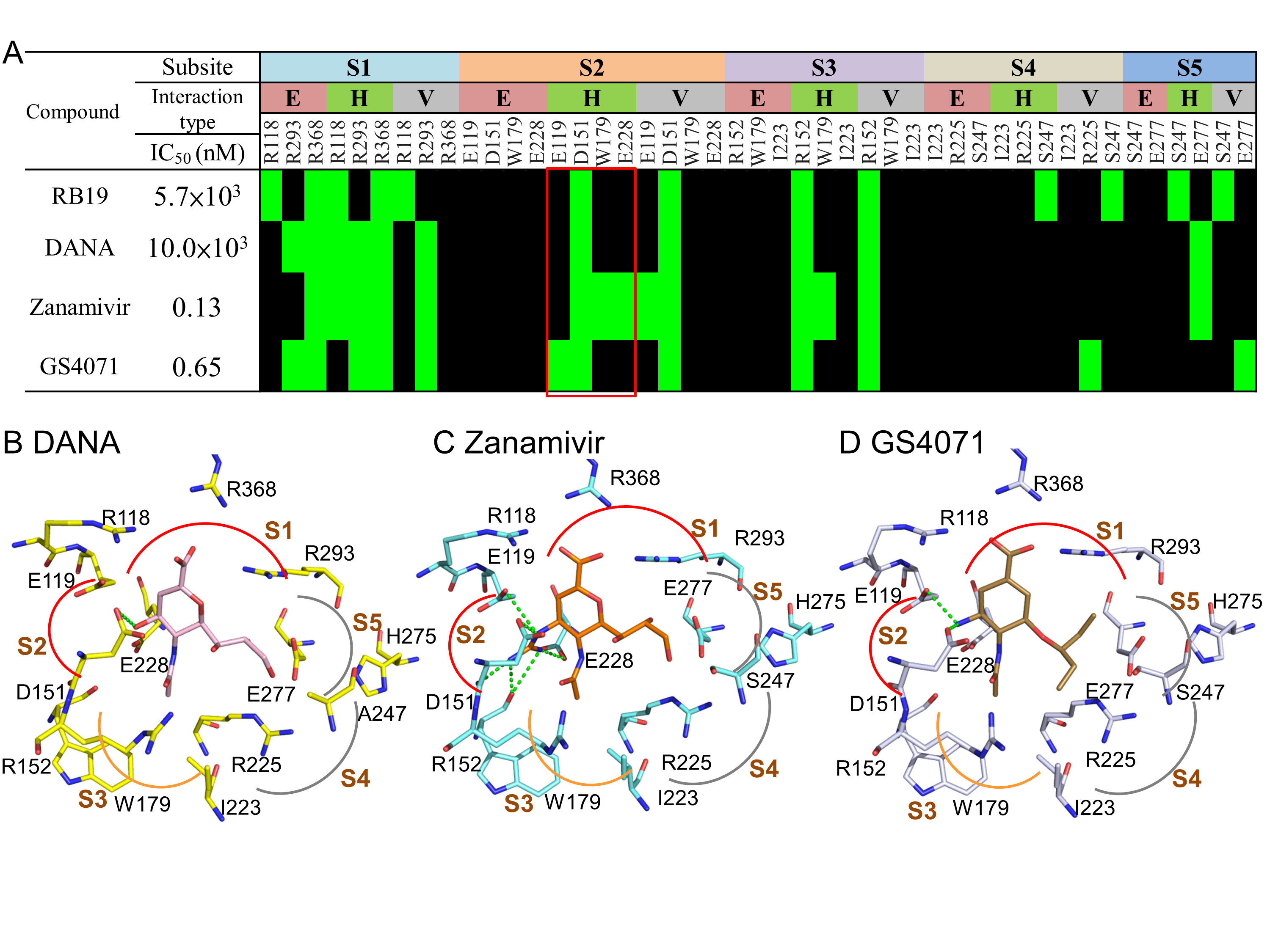

Supplement: Figure S1 — Interaction comparison between RB19, DANA, zanamivir, and GS4071. (A) Interaction profiles between the subsite residues and the compounds. A subsite includes three type interactions (electrostatic (E), hydrogen-bonding (H), and van der Waals (V)). A cell is colored in green if there is interaction (electrostatic, hydrogen-bonding, or van der Waals) between a compound and a residue; otherwise, the cell is colored in black. Binding conformations of (B) DANA (PDB code 1IVF [76]), (C) zanamivir (PDB code 3B7E [37]), and (D) GS4071 (PDB code 2HU4 [67]). The hydrogen-bonding interactions between the compounds and the S2 subsite residues are represented as light green dashes. (TIF) [file pone.0056704.s001.tif]

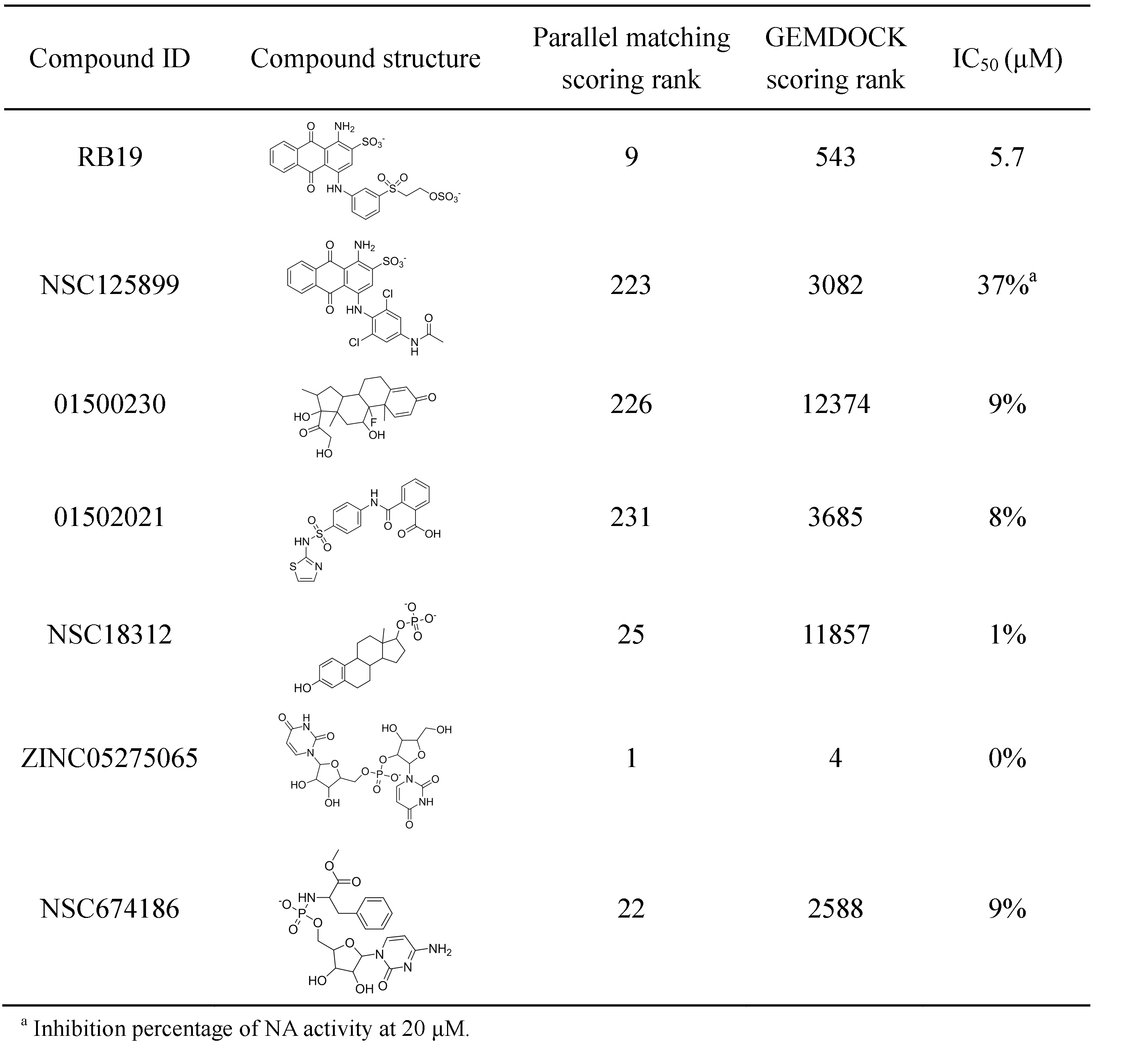

Supplement: Figure S2 — Structures, IC50 values, and ranks of the selected compounds. (TIF) [file pone.0056704.s002.tif]

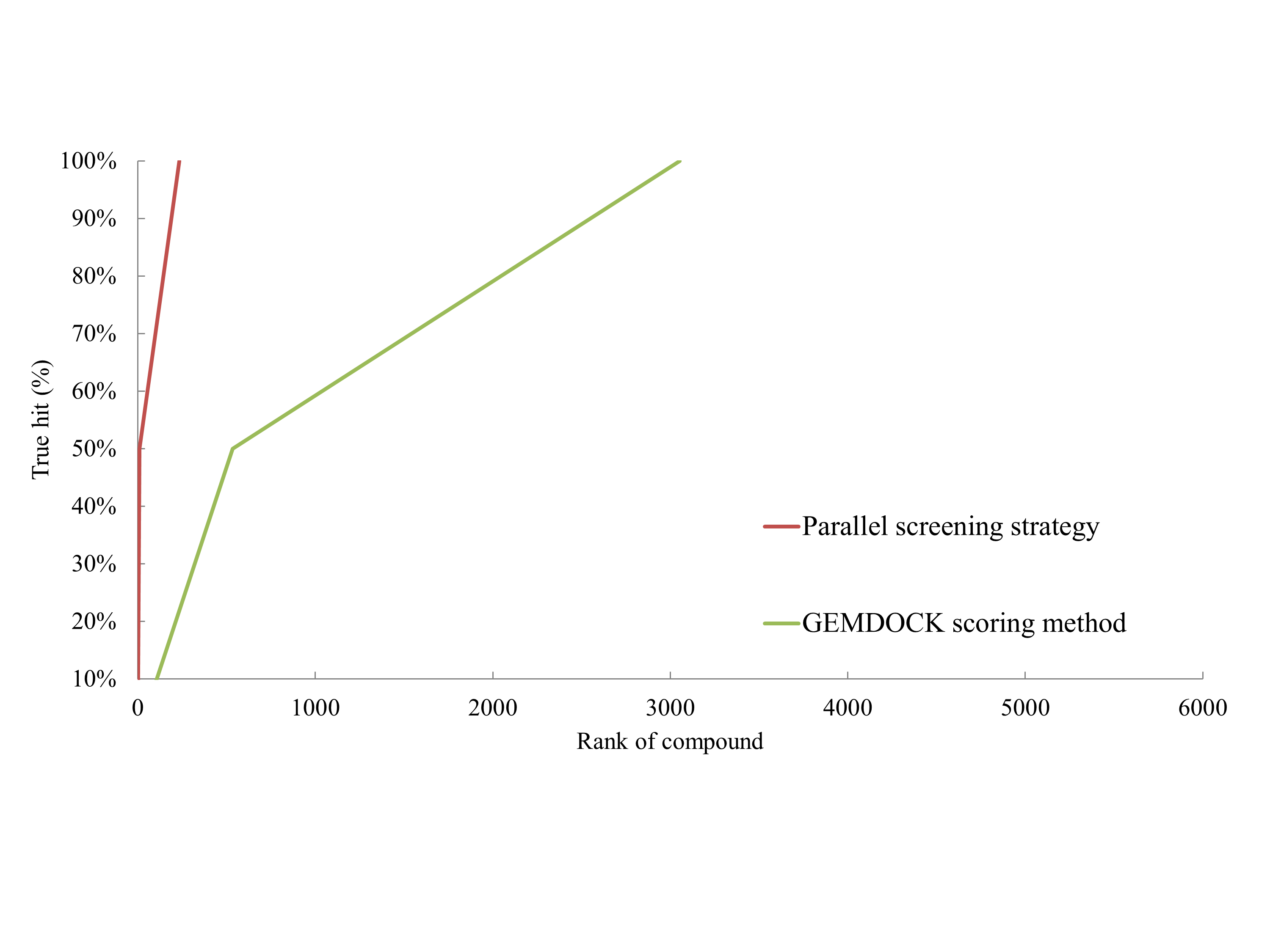

Supplement: Figure S3 — Performance of parallel screening strategy and GEMDOCK scoring method. RB19 and NSC125899 were considered as the hit compounds for comparing the two scoring methods. The parallel screening strategy has better performance than the GEMDOCK scoring method in identifying the hit compounds. For example, RB19 is ranked as 9 and 543 using the parallel screening method and the GEMDOCK scoring method, respectively. (TIF) [file pone.0056704.s003.tif]

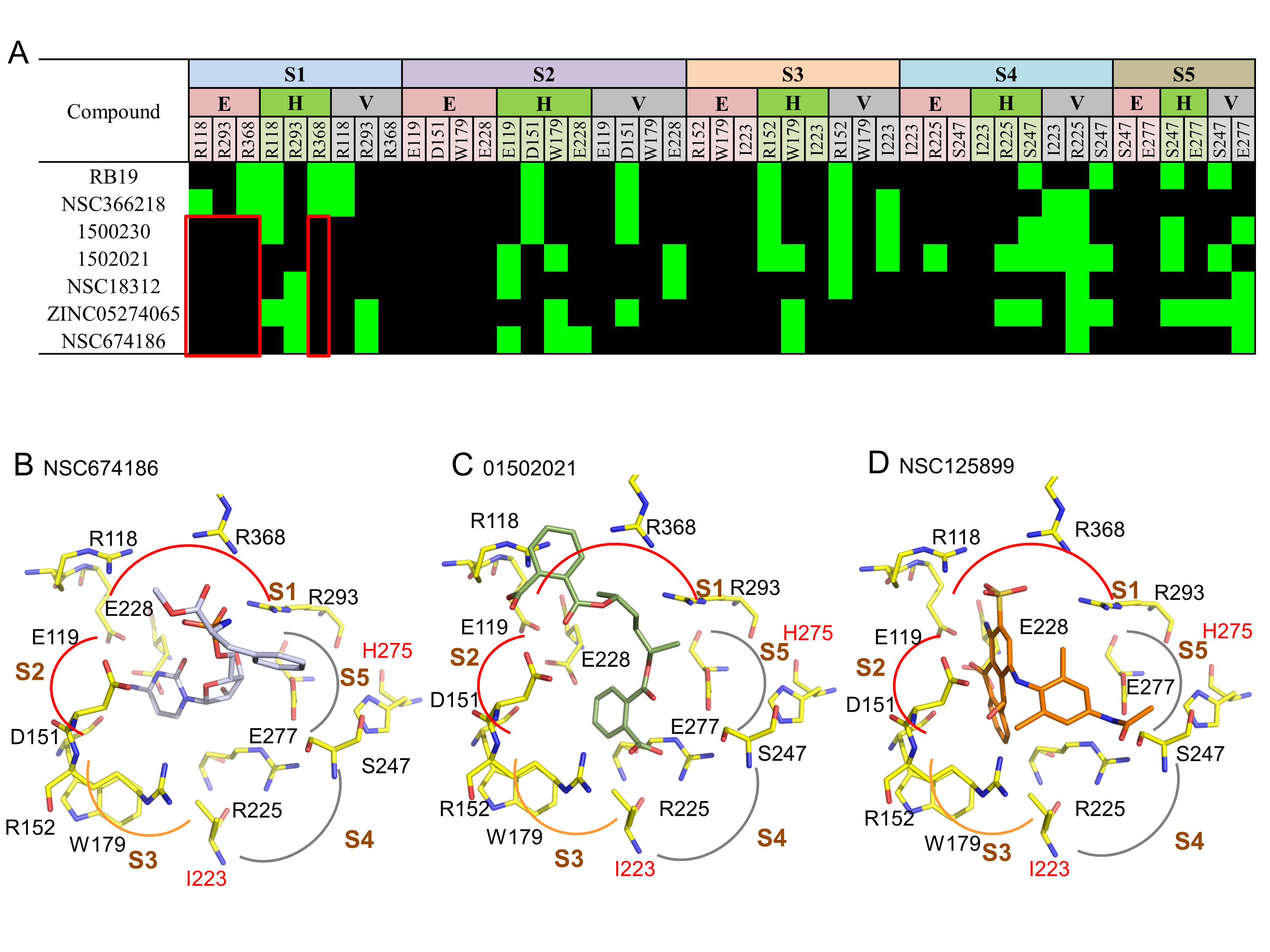

Supplement: Figure S4 — Interaction profiles of seven selected compounds. (A) Interaction profiles between the subsite residues and the compounds. A subsite includes three type interactions (electrostatic (E), hydrogen-bonding (H), and van der Waals (V)) between interaction residues and compounds. A cell is colored in green if a compound forms interaction (electrostatic, hydrogen-bonding, or van der Waals) with a residue; otherwise, the cell is colored in black. Docked conformations of (B) NSC674186, (C) 01502021, and (D) NSC125899 on five subsites. (TIF) [file pone.0056704.s004.tif]

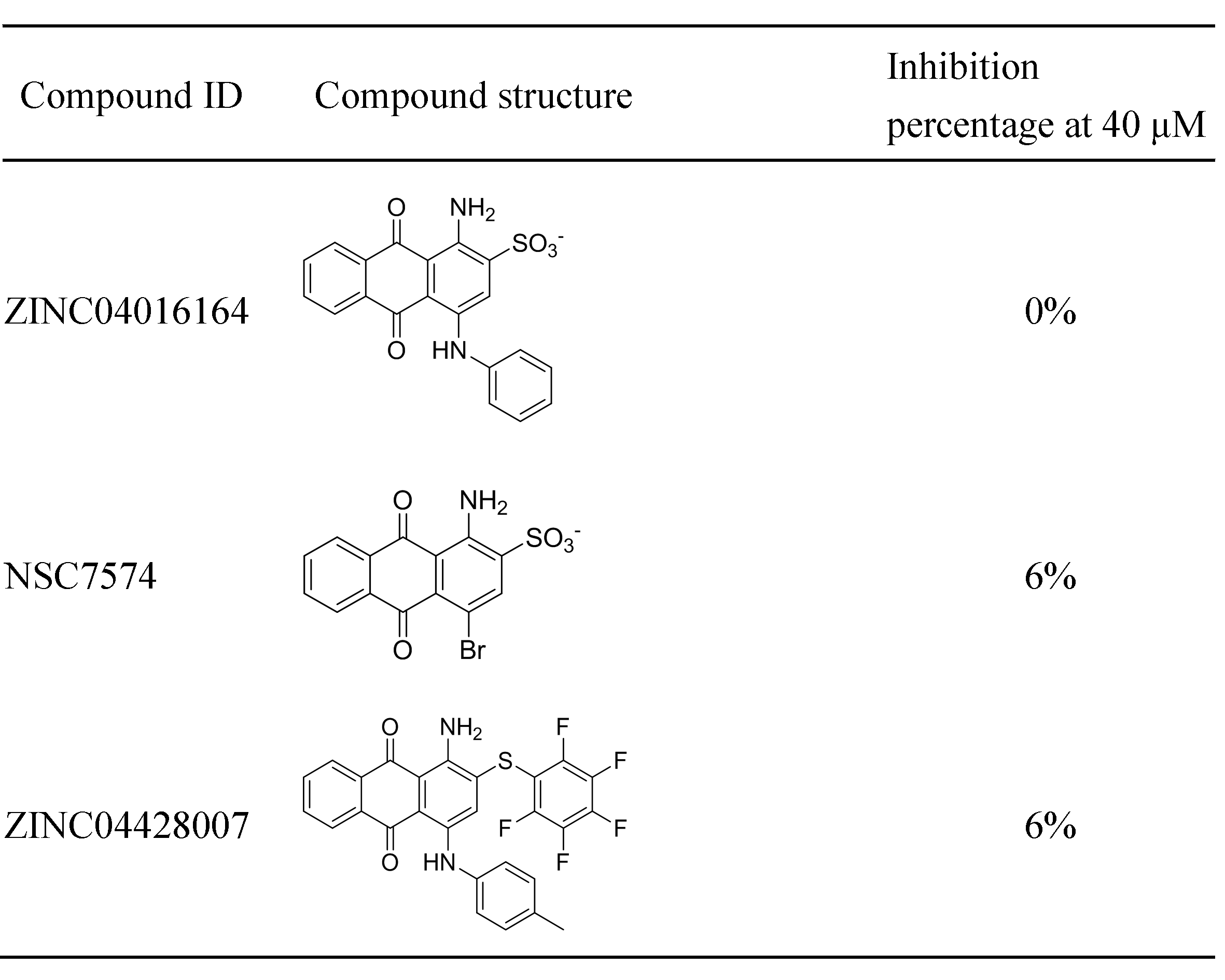

Supplement: Figure S5 — Structures and inhibition percentages of RB19 analogues. (TIF) [file pone.0056704.s005.tif]

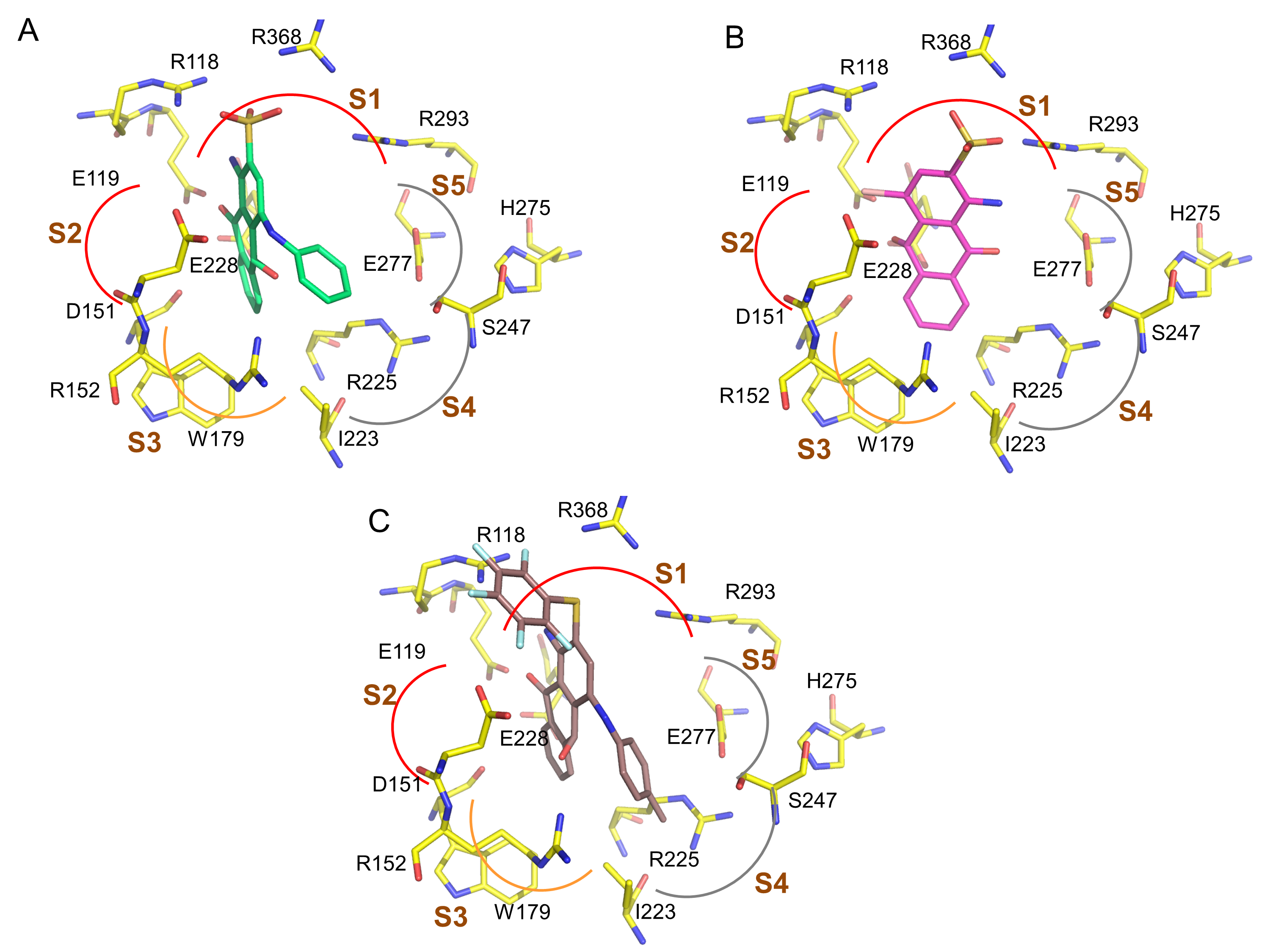

Supplement: Figure S6 — Docking conformations of (A) ZINC04016164, (B) NSC7574, and (C) ZINC04428007 on the wild-type NA of N1. (TIF) [file pone.0056704.s006.tif]

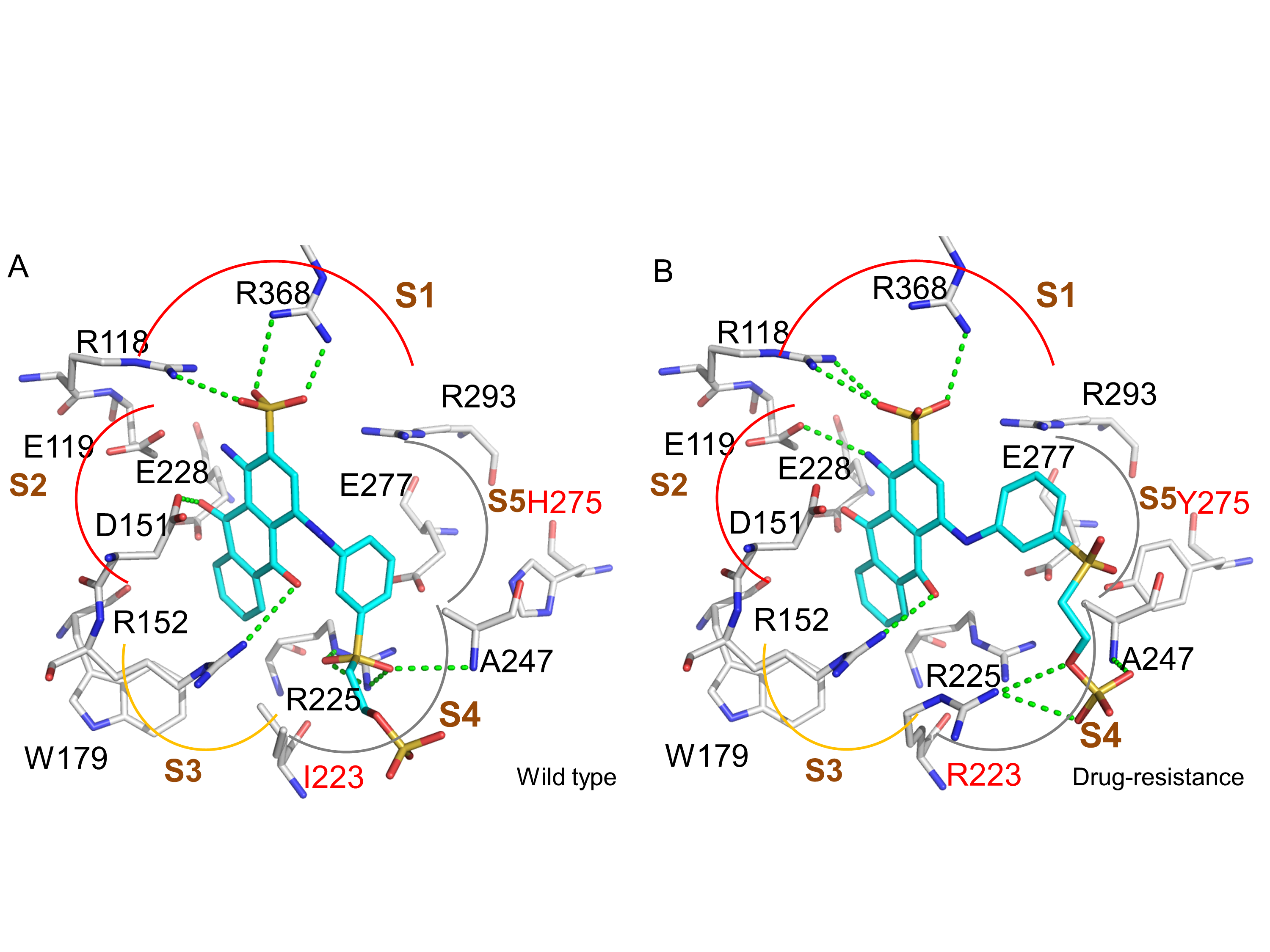

Supplement: Figure S7 — Docking conformations of RB19 on the (A) wild-type NA (PDB code 2AEQ [77]) and (B) the dual H275Y/I223R NA of N2. The structure with the dual mutation of N2 was generated using the similar procedure as the dual-mutant structure of N1. (TIF) [file pone.0056704.s007.tif]
