# Supplementary material for: Parallel Screening of Wild-Type and Drug-Resistant Targets for Anti-Resistance Neuraminidase Inhibitors
Source: PLoS One. 2013 Feb 20;8(2):e56704. doi: 10.1371/journal.pone.0056704 (PMC3577712; doi:10.1371/journal.pone.0056704)
Supplement: Table S1 — Structures, IC50 values, and ranks of the selected compounds. (DOC) [file pone.0056704.s008.doc]

**Table S1.** Structures, IC_50_ values, and ranks of the selected compounds

| Compound ID | Compound structure | Parallel matching scoring rank | GEMDOCK scoring rank | IC_50_ (μM) |
| --- | --- | --- | --- | --- |
| RB19 |  | 9 | 543 | 5.7 |
| NSC125899 |  | 223 | 3082 | 37%^a^ |
| 01500230 |  | 226 | 12374 | 9% |
| 01502021 |  | 231 | 3685 | 8% |
| NSC18312 |  | 25 | 11857 | 1% |
| ZINC05275065 |  | 1 | 4 | 0% |
| NSC674186 |  | 22 | 2588 | 9% |

^a^ Inhibition percentage of NA activity at 20 μM.
