# Supplementary material for: Parallel Screening of Wild-Type and Drug-Resistant Targets for Anti-Resistance Neuraminidase Inhibitors
Source: PLoS One. 2013 Feb 20;8(2):e56704. doi: 10.1371/journal.pone.0056704 (PMC3577712; doi:10.1371/journal.pone.0056704)
Supplement: Table S2 — Structures and IC50 values of RB19 analogues. (DOC) [file pone.0056704.s009.doc]

**Table S2.** Structures and IC_50_ values of RB19 analogues.

| Compound ID | Compound structure | Inhibition percentage at 40 μM |
| --- | --- | --- |
| ZINC04016164 |  | 0% |
| NSC7574 |  | 6% |
| ZINC04428007 |  | 6% |
